# *Agrobacterium rhizogenes*: paving the road to research and breeding for woody plants

**DOI:** 10.3389/fpls.2023.1196561

**Published:** 2023-11-14

**Authors:** Wei Ying, Guangchao Wen, Wenyuan Xu, Haixia Liu, Wona Ding, Luqing Zheng, Yi He, Huwei Yuan, Daoliang Yan, Fuqiang Cui, Jianqin Huang, Bingsong Zheng, Xiaofei Wang

**Affiliations:** ^1^ State Key Laboratory of Subtropical Silviculture, Zhejiang A&F University, Hangzhou, Zhejiang, China; ^2^ Zhejiang Provincial Key Laboratory of Forest Aromatic Plants-based Healthcare Functions, Zhejiang A&F University, Hangzhou, Zhejiang, China; ^3^ College of Science and Technology, Ningbo University, Ningbo, Zhejiang, China; ^4^ College of Life Sciences, Nanjing Agricultural University, Nanjing, Jiangsu, China

**Keywords:** gene function analysis, hairy root, regeneration, Ri plasmid, transformation

## Abstract

Woody plants play a vital role in global ecosystems and serve as valuable resources for various industries and human needs. While many woody plant genomes have been fully sequenced, gene function research and biotechnological breeding advances have lagged behind. As a result, only a limited number of genes have been elucidated, making it difficult to use newer tools such as CRISPR-Cas9 for biotechnological breeding purposes. The use of *Agrobacterium rhizogenes* as a transformative tool in plant biotechnology has received considerable attention in recent years, particularly in the research field on woody plants. Over the past three decades, numerous woody plants have been effectively transformed using *A. rhizogenes*-mediated techniques. Some of these transformed plants have successfully regenerated. Recent research on *A. rhizogenes*-mediated transformation of woody plants has demonstrated its potential for various applications, including gene function analysis, gene expression profiling, gene interaction studies, and gene regulation analysis. The introduction of the Ri plasmid has resulted in the emergence of several Ri phenotypes, such as compact plant types, which can be exploited for Ri breeding purposes. This review paper presents recent advances in A. rhizogenes-mediated basic research and Ri breeding in woody plants. This study highlights various aspects of A. rhizogenes-mediated transformation, its multiple applications in gene function analysis, and the potential of Ri lines as valuable breeding materials

## 1 Introduction

The woodiness proportion among the global vascular plant population ranges from 45% to 48% (FitzJohn et al., 2014). Woody plants play a vital role in meeting human needs by serving as a source of energy, construction materials, and sustenance, while also offering essential ecosystem services such as carbon sequestration, biodiversity support, and climate regulation (Trumbore et al., 2015). Woody plants, encompassing both trees and shrubs, exhibit comparatively lengthier generation times than their herbaceous counterparts, which typically display shorter generation times. Understanding the fundamental biology of woody plants is of utmost importance to enhance their environmental resilience, productivity, and other desirable traits through technological advancements, thus facilitating the cultivation of novel varieties. Among the various methods available, *Agrobacterium*-mediated transformation is the most commonly employed approach. Both *Agrobacterium tumefaciens* and *Agrobacterium rhizogenes* possess the ability to infect plant cells and facilitate the transfer of a DNA segment, referred to as T-DNA, which carries oncogenes from the pathogen to the plant cells. Subsequently, this T-DNA integrates into the genome of the host plant. In the case of *A. tumefaciens*-mediated transformation, there are two obstacles for woody plants: *in vitro* (tissue culture-dependent) manipulation and regeneration from explants. Conversely, *A. rhizogenes*-mediated transformation allows for *ex vitro* (tissue culture-independent) manipulation, resulting in the production of composite plants within a shorter time frame (Meng et al., 2019; Cao et al., 2023). *A. rhizogenes* is a Gram-negative bacterium, which contains the root-inducing (Ri) plasmid that induces plants to produce hairy roots from wounds (Moore et al., 1979; White and Nester, 1980a; Chilton et al., 1982; Lothar et al., 1982). Currently, a wide range of plant species, including angiosperms (both dicotyledonous and monocotyledonous plants), gymnosperms, and even moss, have been found to be susceptible to successful infection by *A. rhizogenes* (De Cleene and De Ley, 1981; Porter and Flores, 1991; Spiess et al, 1977; Masako et al., 2004). The T-DNA fragment on the Ri plasmid integrates into the host plant genome, causing hairy root formation. Plants regenerate from hairy roots, while Ri T-DNA transmits through meiosis (Tepfer, 1984). Therefore, *A. rhizogenes* has become a useful tool for plant biotechnology. The principal steps and factors involved in *A. rhizogenes*-mediated plant transformation are similar to those of *A. tumefaciens* and have been comprehensively examined in previous studies (Gelvin, 2009; Gelvin, 2010; Pitzschke, 2013). It can be suggested that due to similarity in the linear organization of genetic loci that perform equivalent functions during T-DNA transfer, Ti and Ri plasmids appear to be very similar in structure and function in regard to mobilization and transfer of T-DNA (White and Nester, 1980b; Risuleo et al., 1982; Hooykaas et al., 1984; Huffman et al., 1984; Jouanin, 1984). This is confirmed by the genomic sequence of *A. rhizogenes* strain LBA9402 (Hooykaas and Hooykaas, 2021). Four *rol* (for root locus) genes, *rolA*, *rolB*, *rolC*, and *rolD*, have been the object of intense study and are mainly responsible for inducing hairy root formation (Mauro et al., 2017). Analyses have been largely performed in dicotyledonous plants such as tobacco, carrot, tomato, and kiwi, transformed with single genes or in combination (Capone et al., 1989; Christophe et al., 1991; Rugini et al., 1991; Van Altvorst et al., 1992). Auxin synthesis genes, such as *aux1* and *aux2*, collaborate with *rol* genes to facilitate the induction of hairy root formation by supplying auxin. However, the absence of the auxin biosynthetic genes *tms1* and *tms2* in mannopine-type Ri plasmids does not affect root induction, suggesting that Ri plasmids may also alter the development of transformed explants through signaling other than auxin synthesis (Hansen et al., 1991). Opine synthesis genes including *ags* and *mas* can synthesize different opines. The classification of *A. rhizogenes* strains is primarily determined by the specific type of opine they produce. These strains can be divided into four groups: agropine (Petit et al., 1983), mannopine (Petit et al., 1983), mikimopine (Akira et al., 1990), and cucumopine (Elisabeth et al., 1988). According to the opine genes present in Ri plasmids, more than 20 strains of *A. rhizogenes* used for genetic transformation have been assigned to these aforementioned categories (Bahramnejad et al., 2019).

Almost three decades after the initial successful transformation of *A. rhizogenes* in grapevine (Guellec et al., 1990), numerous successful transformations have been performed in woody plants, typically in the form of hairy roots or composite plants. In addition, the regeneration of transgenic hairy roots in various plants has further demonstrated the applicability of the *A. rhizogenes* transformation system in molecular plant breeding. Hairy roots cultivated *in vitro* serve as a primary means for the synthesis of secondary metabolites, particularly within medicinal plants (Sharma et al., 2013). Furthermore, the utilization of hairy roots and composite plants in scientific investigations to explore gene functionality is extensive, encompassing various aspects such as root development, wood formation (Plasencia et al., 2016), interactions between roots and soil microbes (Plett et al., 2014), and plant allelopathy (Stanisic et al., 2019). This review aims to delve into the process of transformation of woody plants through the application of *A. rhizogenes*, while also discussing the potential implications of such transformations for basic research and biotechnological advancement.

## 2 Agrobacterium rhizogenes as a root agent

The propagation of plants through stem cuttings is commonly used in the commercial production of ornamental plants, medicinal plants, and timber trees. However, some commercially important tree species exhibit a low root formation rate. As far back as 1930, the induction of hairy roots was observed in nursery apple trees (Riker et al., 1930), and it was subsequently determined that the root induction was caused by *A. rhizogenes* (Hildebrand, 1934). Since then, *A. rhizogenes* has been used to enhance root formation in plants that are difficult to propagate through stem cuttings (Zavattieri et al., 2016).

The use of *A. rhizogenes* as a rooting inducer for difficult-to-root plants has been found to be remarkably effective and suitable for a wide range of plants. The determination of the root-promoting ability of *A. rhizogenes* is contingent upon the combination of bacterial strains and plant genotypes. Within woody plant species, there exists variation in rooting efficiency. Additionally, the specific strain of *A. rhizogenes* employed is a critical factor. For instance, in the case of the challenging-to-root woody species, “Golden Delicious” apple, the induction of rooting rates ranges from 0% to 20%, depending on the strain of *A. rhizogenes* utilized. Notably, strains A4 and 232 successfully induce adventitious root formation, whereas strains 178 X A4T and R1000 are unable to do so (Pateña et al., 1988). Cuttings from mature jujube trees (*Ziziphus jujuba* Mill.) exhibit a significant challenge in rooting. However, the application of *A. rhizogenes*, specifically strain TR105, resulted in the highest root formation percentage (65%), which was twice as high as that of the uninoculated cuttings (32.5%). Conversely, strain A4 did not show any significant difference in root formation (Mochammad et al., 1996). Interestingly, in the case of *Corylus avellana*, the rooting rate reached 100% when inoculated with a combination of *A. rhizogenes* (A7 + 22) (Bassil et al., 1991). In addition to bacterial strains, the success of inoculation and production of hairy roots is significantly influenced by plant genotypes and states, as evidenced by multiple studies. For instance, in hazelnut, the stimulation of rooting of cuttings was found to be influenced by both the cultivar and the date of cutting collection (Bassil et al., 1991). Similar results have also been observed in other research groups (Magnussen et al., 1994; Mihaljevic et al., 1996; Sarmast et al., 2019). The infectivity and adventitious root production of *A. rhizogenes* in host plant tissue are contingent upon the compatibility between *A. rhizogenes* and host plants, the responsiveness of the plant tissues to the T-DNA, the production of phytohormones, and the juvenile state of the host tissues.

Auxin is a well-known root inducer, and exogenously applied auxins have been shown to accelerate the rooting process in cuttings of a wide variety of plant species. When auxin and *A. rhizogenes* are combined, they exhibit varying effects on branch rooting, including synergy, antagonism, or no effect. Previous studies have observed a synergistic action between IBA and *A. rhizogenes* in inducing rooting in radiata pine (Li and Leung, 2003) and walnut (Caboni et al., 1996). Conversely, an antagonistic action between IAA and *A. rhizogenes* has been observed in inducing rooting in *Pinus monticola* (McAfee et al., 1993). The combined effect of *A. rhizogenes* to stimulate rooting is contingent upon the specific species and genotypes of micro propagated fruit trees. In all tested genotypes, root formation was observed following infection with *A. rhizogenes*. Three distinct responses were observed: genotypes that rooted without the presence of auxins showed a decrease in rooting percentage when auxin and infection were combined; genotypes that rooted only with auxin exhibited either no effect or a synergistic effect between auxins and infection; genotypes that rooted solely with *A. rhizogenes* displayed either no effect or an antagonistic effect between auxins and infection (Carmine et al., 1998). According to Zarei et al. (2020), the induction of rooting in *Picea abies*, a species known for its reluctance in rooting, cannot be achieved solely through the use of *A. rhizogenes*. However, when *A. rhizogenes* is combined with auxin, successful rooting can be achieved (Zarei et al., 2020).

A histological investigation revealed that the development of auxin-induced roots differs from that of *A. rhizogenes*. In the presence of NAA, adventitious roots are generated endogenously, originating within the vascular tissues of the stem. Conversely, adventitious roots formed in response to *A. rhizogenes* infection exhibit both endogenous and exogenous growth patterns. In the process of endogenous root formation, calli are generated within the cortex, leading to the subsequent formation of tracheid nests, which results in a bulge in the stem. Additionally, exogenous callus, formed at the base of shoot, also gives rise to tracheid nests. Consequently, roots form from both of these callus structures (Ellen and Juvenal, 1993). The rooting process in walnut was found to be influenced by the combined action of IBA and *A. rhizogenes*, as well as the antagonistic effect of IAA and *A. rhizogenes*. Notably, a significant reduction in the roots containing bacteria was observed when *A. rhizogenes* was combined with either IAA or IBA (Falasca et al., 2000).

It is imperative to conduct strain screening to optimize the rooting rate for each specific plant species. The utilization of multiple strains of *A. rhizogenes* offers a captivating approach to enhance rooting. The modulation of plant sensitivity to auxin by *A. rhizogenes* is believed to contribute to the promotion of rooting (Shen et al., 1988; Spanò et al., 1988; Petersen et al., 1989; Christophe et al., 1991). This modulation is thought to occur through variations in endogenous auxin levels and auxin sensitivities across different plant species (Carmine et al., 1998).

## 3 Agrobacterium rhizogenes as genetic engineers

### 3.1 Transformation

*A. rhizogenes* not only facilitates the rooting of difficult-to-propagate plants, but also allows for the integration of foreign genes into the plant genome through binary vectors. Researchers have successfully transferred foreign genes into various woody plant species, including *Larix decidua* (Huang et al., 1991), *Alhagi pseudoalhagi*, *Eucalyptus camaldulensis* (Balasubramanian et al., 2011), *Prunus* (Bosselut et al., 2011; Xu et al., 2020), *Parasponia* (Cao et al., 2012), *Trema* (Cao et al., 2012), *Poncirus trifoliata* (Xiao et al., 2014), *Solanum erianthum* (Sarkar et al., 2020), *Populus* (Neb et al., 2017), *Salix purpurea* (Gomes et al., 2019), *Malus prunifolia* (Yamashita et al., 2004), and *Litchi chinensis* (Qin et al., 2021), by infecting plant organs such as cotyledons, hypocotyls, stem segments, root segments, leaves, petioles, callus, and *in vitro* shoots with *A. rhizogenes* (**Table 1**). The use of *in vitro* shoots as explants has enabled the development of composite plants, which have numerous biological applications, such as nutrient absorption, biotic and abiotic stress tolerance, and signal exchange between aboveground and underground plant parts. However, the tissue culture of woody plants presents several technical challenges, such as browning and the need for aseptic conditions, which require skilled operators and the identification of suitable bacteriostatic agents for different strains and explants. To address these challenges, Collier et al (2005) developed a novel method to induce *A. rhizogenes* infection in plants without the use of tissue culture, thereby generating composite plants (Collier et al., 2005). This approach not only is cost-effective and efficient, but also enables the production of composite plants in a short time frame without the need for tissue culture. Furthermore, it is simple to execute, boasts a short cycle, and does not require complex sterilization procedures. Collier and colleagues successfully applied this technique to 14 dicotyledonous herbs from nine genera spanning four families. Following infection with the same strain, 14 composite dicotyledonous plants were generated, with transformation efficiencies ranging from 56% to 100% (Collier et al., 2005). This method has also been successfully implemented in woody plants. Using seedlings as explants, composite plants have been generated from a wide range of woody plants, including *Camellia sinensis* (Alagarsamy et al., 2018), *Discaria trinervis* (Imanishi et al., 2011), *Persea americana* (Prabhu et al., 2017), *Taxus baccata* (He et al., 2022), *Carica papaya* (Hoang et al., 2022), *Citrus* (Ma et al., 2022), *Ailanthus altissima* (Cao et al., 2023), *Aralia elata* (Cao et al., 2023), *Clerodendrum chinense* (Cao et al., 2023), *Caragana sinica* (Diouf et al., 1995), and *Malus pumila* (Pawlicki-Jullian et al., 2002; Yamashita et al., 2004) (**Table 1**). Although seedlings can be used as explants for woody plants, the genetic heterozygosity of these plants requires the use of a sufficient number of transformed lines to ensure the accuracy of experimental results, particularly in studies of stress resistance and other biological phenomena. In addition, stem cutting represents a crucial means of reproducing woody plants. In this regard, Ma et al (2022) successfully generated composite plants using citrus stem cuttings as explants, resulting in composite plants that possess the same genetic background, and are therefore more suitable for biological research applications (Ma et al., 2022).

**Table 1:** Woody plants transformed by *A. rhizogenes*.

Plant species	Infected explants	*A. rhizogenes* strain(s)	Infected condition	Transformants	Regeneration pathway	References
*Actinidia deliciosa*	Cuttings	NIAES 1724	*In vitro*	Transgenic plants	Organogenesis*	Yazawa et al., 1995
*Aesculus hippocastanum*	Androgenic embryos	A4GUS	*In vitro*	Transgenic plants	Organogenesis	Zdravkovic-Korac et al., 2004
*Ailanthus altissima*, *Aralia elata*, *Clerodendrum chinense*	Seedlings	K599	*Ex vitro*	Transgenic plants	Organogenesis	Cao et al., 2023
*Alhagi pseudoalhagi*	Cotyledon and hypocotyl	A4	*In vitro*	Transgenic plants	Organogenesis	Wang et al., 2001
*Allocasuarina verticilkita*	Epicotyl, cotyledon, and hypocotyl	A4, 2659	*In vitro*	Transgenic plants	Organogenesis*	Phelep et al., 1991
*Aralia elata*	Petiole, roots, leaves	ATCC 15834	*In vitro*	Transgenic plants	Somatic embryogenesis	Kang et al., 2006
*Cajanus cajan*	Root, hypocotyl, stem, cotyledon, leaves, and petiole	ATCC43057, R1601, LBA9402, A4, ATCC15834	*In vitro*	Hairy root		Jiao et al., 2020
*Camellia assamica*	Leaves	LBA9402	*In vitro*	Hairy root		Tisserant et al., 2016
*Camellia sinensis*	Callus	ATCC15834	*In vitro*	Hairy root		Rana et al., 2016
*Camellia sinensis*	Seedlings	A4	*Ex vitro*	Composite plants		Alagarsamy et al., 2018
*Caragana sinica*, *Aquilaria sinensis*, *Malus domestica*, *Malus hupehensis*, *Malus pallasiana*	Seedings	K599	*In vitro* and *ex vitro*	Transgenic plants	Organogenesis	Wu et al., 2012
*Carica papaya*	Leaves	LBA9402	*In vitro*	Transgenic plants	Somatic embryogenesis	Cabrera-Ponce et al., 1996
*Carica papaya*	Hypocotyl	K599	*Ex vitro*	Composite plants		Hoang et al., 2022
*Casuarina glauca*	Seedlings	A4RS	*In vitro*	Composite plants		Diouf et al., 1995
Cherry rootstock Colt	Shoots	NCPPB 1855	*In vitro*	Transgenic plants	Somatic embryogenesis	Gutierrez-Pesce et al, 1998
*Citrus*	Stem cuttings	K599	*Ex vitro*	Composite plants		Ma et al., 2022
*Citrus*	Seedlings	ATCC 43056	*In vitro*	Transgenic plants	Organogenesis	Ramasamy et al., 2023
*Coffea arabica*	Roots, hypocotyls, cotyledons	A4RS, Rqua1, 1724, 2659, 8196	*In vitro*	Composite plants		Alpizar et al., 2006
*Coffea canephora*	Seedlings	A4	*In vitro*	Transgenic plants	Somatic embryogenesis	Kumar et al., 2006
*Discaria trinervis*	Seedlings, stem cuttings	A4RS, ARqua1	*In vitro* and *ex vitro*	Composite plants		Imanishi et al., 2011
*Dryas drummondii*, *Dryas octopetala*	Seedlings	AR1193	*In vitro*	Composite plants		Billault-Penneteau et al., 2019
*Duboisia myoporoides* x *D. leichhardtii*	Leaves	A4	*In vitro*	Transgenic plants	Organogenesis*	Roig Celma et al., 2001
*Eucalyptus camaldulensis*	Seedlings	A4RS	*In vitro*	Composite plants		Bosselut et al., 2011
*Eucalyptus grandis*	Seedlings	A4RS	*In vitro*	Hairy root and composite plants		Plasencia et al., 2016
*Ginkgo biloba*	Zygotic embryos	A4	*In vitro*	Hairy root		Ayadi and Trémouillaux-Guiller, 2003
*Justicia gendarussa*	Leaf petiole	A4 and MTCC 532	*In vitro*	Hairy root		Largia et al., 2022
*Larix decidua*	Hypocotyl	11325	*In vitro*	Transgenic plants	Organogenesis	Huang et al., 1991
*Litchi chinensis*	Leaves and stem segments	MSU440	*In vitro*	Hairy root		Qin et al., 2021
*Malus baccata*	Shoots	8196	*In vitro*	Transgenic plants	Organogenesis	Wu et al., 2012
*Malus pumila*	Shoots	A4	*In vitro*	Transgenic plants	Organogenesis	Lambert and Tepfer, 1992
*Malus pumila*	Shoots	8196, A4, 15834	*In vitro*	Transgenic plants	Organogenesis	Pawlicki-Jullian et al., 2002
*Malus pumila*	Stem	MAFF 02-10266, 03-01724, 03-01725	*In vitro*	Transgenic plants	Organogenesis	Yamashita et al., 2004
*Morus indica*	Seedlings	MAFF 210268 and 720001, MAFF 210265	*In vitro*			Oka and Tewary, 2000
*Parasponia andersonii*, *Trema tomentosa*	Shoots	MSU440	*In vitro*	Composite plants		Cao et al., 2012
*Persea americana*	Seedlings	K599 or ARqua1	*Ex vitro*	Composite plants		Prabhu et al., 2017
*Pinus contorta*	Seedlings	LBA 9402, A4RSII	*In vitro*	Composite plants		Yibrah et al., 1996
*Poncirus trifoliata*	Leaves, epicotyls	MSU440, K599	*In vitro*	Transgenic plants	Organogenesis	Xiao et al., 2014
poplars	Shoot cuttings	1724, K599, 8196, 15834	*In vitro*	Composite plants		Neb et al., 2017
*Prunus persica*	Cuttings	A4R	*In vitro*	Composite plants		Bosselut et al., 2011
*Prunus persica*	Hypocotyl, leaves, seedlings	MSU440	*In vitro*	Hairy root and composite plants		Xu et al., 2020
*Punica granatum*	radicle, cotyledon, leaves	MSU440, A4, 15834	*In vitro*	Hairy root		Ono et al., 2012
*Rauwolfia serpentina*	Leaves	A4	*In vitro*	Transgenic plants	Organogenesis*	Mehrotra et al., 2013
*Robinia pseudoacacia*	Hypocotyls	RI601	*In vitro*	Transgenic plants	Organogenesis	Han et al., 1993
*Salix purpurea* and *Salix* spp.	Shoots	A4RS	*In vitro*	Hairy root and composite plants		Gomes et al., 2019
*Solanum erianthum*	Leaves	A4	*In vitro*	Hairy root		Sarkar et al., 2020
*Taxus baccata*	Seedlings, shoots	A4	*In vitro* and *ex vitro*	Composite plants		He et al., 2022

*Automatically organogenesis from hairy root.

### 3.2 Impact factors

The core factors that impact the transformation efficiency of *A. rhizogenes* are the same as those that influence root growth promotion when *A. rhizogenes* is employed as a rooting agent. These factors include the type of *A. rhizogenes* strains and the genetic or genotype or states of the host plants. For instance, in the context of poplar stem segment transformation, the four strains (1724, K599, 8196, and 15834, representing mikimopine, cucumopine, mannopine, and agropine strains, respectively) exhibit varying differences in hairy root formation time, number of formations, and transformation efficiency. Hence, it is essential to identify suitable strains for specific plants (Neb et al., 2017). Moreover, variations can also arise between species, varieties, and clones. For instance, a study involving five woody plants found that *Aquilaria sinensis* failed to produce hairy roots, while the remaining five plants generated composite plants with efficiencies ranging from 30% to 85% (Meng et al., 2019). In 22 citrus species, transformation efficiencies fluctuated from 0 to 95% (Ma et al., 2022).

Other factors, such as the tissue and physiological conditions of explants, bacterial concentration, acetosyringone concentration, hormone recipes, and additional treatments such as vacuuming will affect transformation efficiency. Leaves, petioles, stem segments, and adventitious buds can be used as explants. Suitable explants vary from plant to plant. For example, in *Cajanus cajan*, leaves have the highest transformation efficiency, reaching 70.92% (Jiao et al., 2020). In *L. chinensis*, there is no significant difference between the transformation efficiency of stem segments and leaves (Qin et al., 2021). In *Coffea arabica*, hypocotyls of cultivars of Caturra and IAPAR-59 had the highest infection efficiencies, reaching 82% and 51%, respectively (Alpizar et al., 2006). The age of the plants also affects the transformation efficiency; the transformation efficiency of 8-week-old seedlings was significantly higher than that of 20-week-old *P. americana* (Prabhu et al., 2017). After *A. rhizogenes* infection, hairy roots are often produced through cortical cells (Falasca et al., 2000). The older the physiological age, the higher the degree of lignification, the lower the proportion of cortical cells, and the longer the time used for hairy root induction. In addition, the concentration of the bacterial solution also affects transformation efficiency. In general, the concentration of *A. rhizogenes* infecting plants is between 0.4 and 1.0 (Ma et al., 2022; Cao et al., 2023). At the same time, full induction of the *vir* gene in the strain requires acetosyringone, a chemoattractant for *Agrobacterium*, at a commonly used concentration of 100–200 µM (Ma et al., 2022; Cao et al., 2023). To improve the transformation efficiency, additional treatments such as vacuum treatment have been applied to facilitate the infiltration *A. rhizogenes* into plant cells (Ma et al., 2022).

### 3.3 Regeneration

**Figure 1:**
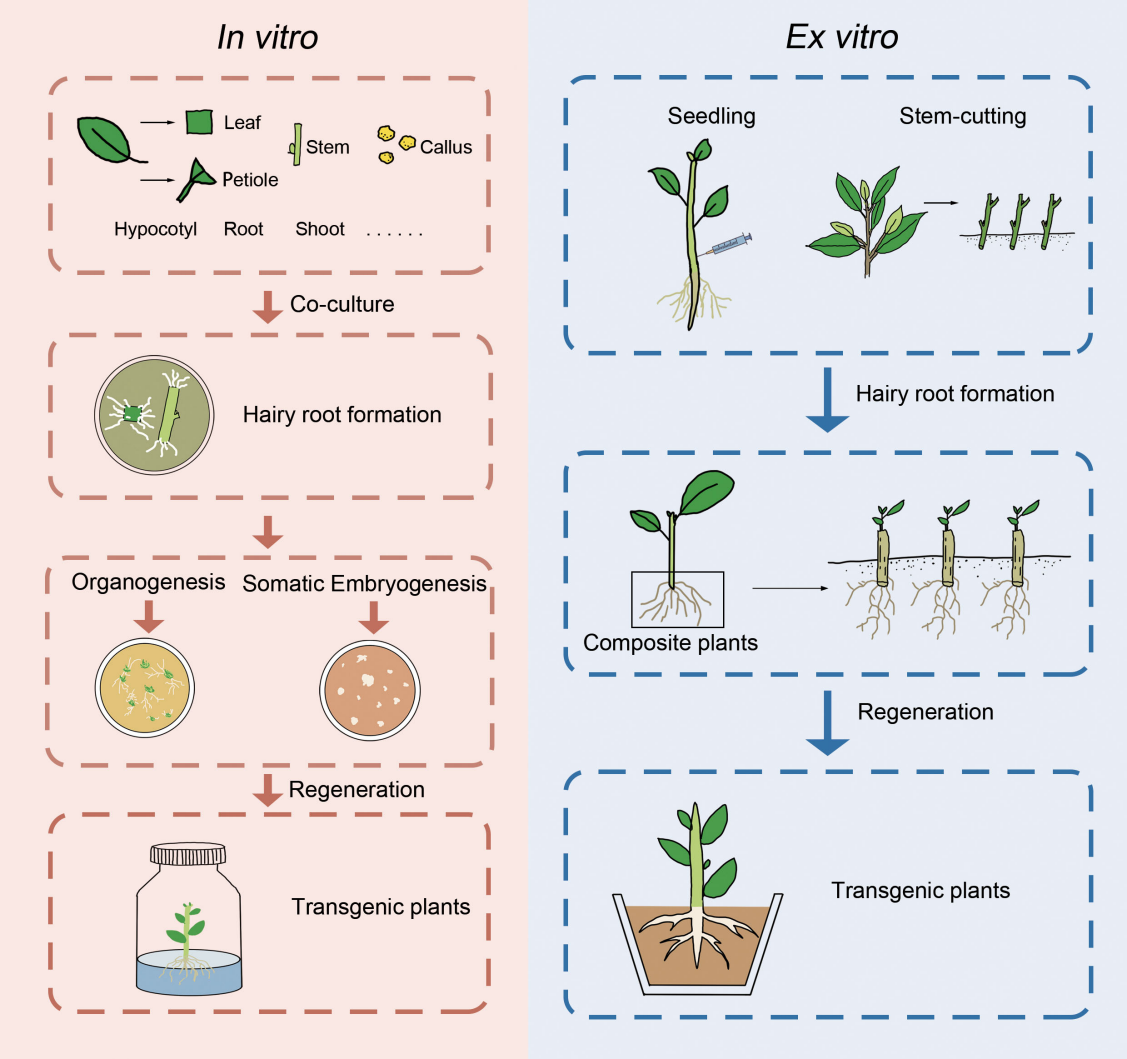
Presentation of *in vitro* and *ex vitro* genetic transformation of woody plants mediated by *A. rhizogenes*.

Hairy roots induced by A. rhizogenes have the potential to facilitate the generation of transgenic plants through various ways, including spontaneous organ regeneration, organogenesis, or somatic embryogenesis (**Figure 1**). Nevertheless, many woody species were induced by regeneration plants via organogenesis or somatic embryogenesis. Some woody plants, such as *Actinidia deliciosa* (Yazawa et al., 1995), *Allocasuarina verticillate* (Phelep et al., 1991), *Duboisia myoporoides x D. leichhardtii* (Trovato et al., 2001), and *Rauwolfia serpentina* (Mehrotra et al., 2013), are capable of regeneration via spontaneous organ regeneration from their hairy roots, which can be completed in hormone-free medium. On the other hand, other woody plants such as *Aesculus hippocastanum* (Zdravkovic-Korac et al., 2004), *A. pseudoalhagi* (Wang et al., 2001), *Larix decidua* (Huang et al., 1991), *M. pumila* (Pawlicki-Jullian et al., 2002), *P. trifoliata* (Xiao et al., 2014), *Robinia pseudoacacia* (Han et al., 1993), and citrus (Ramasamy et al., 2023) require hormone ratios to induce the production of calli, from which regenerated plants can be obtained. It is worth noting that in the study of *Malus baccata*, regenerated plants were only obtained when the hairy roots remained attached to the mother plant (non-transformed aerial part) (Wu et al., 2012). In addition to organogenesis, some woody plants can also produce somatic embryos through the induction of hairy roots, which germinate into regenerated plants. Examples of such plants include *A. elata* (Kang et al., 2006), *C. papaya* (Cabrera-Ponce et al., 1996), cherry rootstock *Colt* (Gutierrez-Pesce et al., 1998), and *Coffea canephora* (Kang et al., 2006). However, despite multiple attempts, some studies have failed to obtain regenerated plants through hairy roots. Recently, a cut-dip-budding method has been successfully applied to three woody plants, namely, *A. altissima*, *A. elata*, and *C. chinense*, which allowed the generation of *A. rhizogenes*-mediated transgenic plants (Cao et al., 2023) (**Figure 1**). The regeneration of woody plants from roots is a common phenomenon and can be enhanced by pruning aboveground parts (Wan et al., 2006). These approaches would facilitate woody plant transformation mediated by *A. rhizogenes*.

## 4 Applications in woody plants

### 4.1 Basic biological research

Currently, *A. tumefaciens*-mediated genetic transformation systems have been successfully implemented in several woody species, such as poplar (Wang et al., 2011), apple (Schropfer et al., 2022), kiwifruit (Uematsu et al., 1991), and walnut (McGranahan et al., 1988). However, the number of woody plants that have been sequenced far exceeds the number of plants that have established transformation systems. With advancements in sequencing technology and the enhanced level of genome assembly, there is a growing potential for the sequencing of numerous genetically intricate woody plant genomes. An example of this progress is the successful completion of the Chinese pine (*Pinus tabuliformis*) genome, which has been assembled at the chromosome level and spans a size of 25.4 gigabases (Gb) (Niu et al., 2022). Consequently, effective use of the substantial amount of sequence information will become an imperative endeavor. Transgenic technology for resolving gene function is emerging as a key tool to address this challenge. In recent years, the utilization of *A. rhizogenes* for the production of composite plants has gained significant traction in the realm of herbaceous plant investigation, particularly in the case of soybean. This development has provided a promising avenue for exploring woody plant research.

The application of *A. rhizogenes*-mediated transformation in soybean has proven to be highly successful in various biological contexts. A particularly efficient and rapid method for this transformation has been established (Kereszt et al., 2007). In soybean research, three main types of applications have been extensively utilized. These include the construction of composite plants through overexpression, RNAi, and CRISPR-Cas9 binary vectors for the purpose of gene function analysis (Traubenik et al., 2020; Li et al., 2021). Additionally, basic molecular analyses, such as promoter analysis (Yang et al., 2021), subcellular localization studies (Brear et al., 2020), ChIP-PCR (Pi et al., 2019), GST pull-down assays (Wang et al., 2015), Co-IP assays (Vadivel et al., 2021), protein ubiquitination and degradation studies (Zhang et al., 2021), and *in vivo* kinase assays (Gao et al., 2022), have been conducted. Of particular interest is mutant complementation (Feng et al., 2021; Jiang et al., 2021), which serves as a genetic validation of gene function.

A variety of studies have used *A. rhizogenes*-mediated transformation technology for basic biological investigations in woody plants, including binary vector generation of composite plants, basic molecular analysis, and genetic analysis. *A. rhizogenes* possesses the ability to carry binary vectors, which facilitates gene overexpression, RNAi, and gene editing. These transformants have been utilized to examine nutrient uptake, abiotic stresses, nodule development, mycorrhizal interactions, allelopathy, biosynthesis, and wood formation. For instance, in apple, the function of the *MdPRP6* gene was elucidated under low nitrogen conditions through *A. rhizogenes*-mediated knockout or overexpression (Zhang et al., 2022). In *Eucalyptus*, the STOP-like gene was disrupted to assess its aluminum resistance function (Sawaki et al., 2014). In poplar, *A. rhizogenes*-mediated transformation yielded *PtJAZ6* knockout and overexpression materials, which were subsequently employed to analyze the mutualistic interaction between *PtJAZ6* and effectors in ectomycorrhizal *Laccaria bicolor* (Plett et al., 2014). This technology has also been applied to *R. pseudoacacia* to identify *Rpf41* as a critical regulator of symbiotic nodulation in legumes (Chou et al., 2016). In apple, the function of *BvSTI* in allelopathy was examined using the hairy root system (Stanisic et al., 2019). Although genetic transformation of tea plants poses challenges, the function of *CsTSI* in theanine biosynthesis was successfully investigated using the *A. rhizogenes* system (She et al., 2022). Furthermore, it is worth noting that woody plant roots, similar to stems, undergo secondary growth, making them a valuable resource for investigating the process of wood formation (Plasencia et al., 2016).

Gene function studies involve analyzing tissue expression, protein interactions, and downstream target genes. The use of hairy roots enables researchers to conduct related research within homologous species, thereby providing a more accurate reflection of gene function. For instance, in citrus, the *CsSUC2* promoter was analyzed in hairy roots (Roig Celma et al., 2001), while in Eucalyptus, the *EgCCR1* and *EgCAD2* promoters were studied in hairy roots (Rugini et al., 1991). Bimolecular fluorescence complementation (BiFC) was used to verify the interaction between *CcCIPK14* and *CcCBL1* in hairy roots of cowpea (Mehrotra et al., 2013). Furthermore, hairy roots can be utilized to screen downstream target genes of transcription factors. For instance, downstream target genes of *MYB15* were identified in grape (White et al., 1985). In poplar, the *A. rhizogenes* system can be used to verify the downstream target genes of transcription factors through ChIP-PCR (Schmülling et al., 1988; Filippini et al., 1996). It is worth noting that genetic manipulation of woody plants, especially the genetic analysis of upstream and downstream genes, is challenging. The use of the *A. rhizogenes* system has overcome this challenge in some species. For example, Ma (2018) used *A. rhizogenes* to knock out *MdSUT2.2* based on *MdCIPK22* transgenic apple plants, which confirmed that *MdCIPK22* depends on *MdSUT2.2* for drought tolerance (Zhu et al., 2003).

### 4.2 Ri breeding

Plants regenerated from hairy roots exhibit phenotypes, including vigorous large root growth, lateral root development, root geotropism loss, loss of shoot apical dominance, internode shortening, and plant dwarfing (Han et al., 1993; Yazawa et al., 1995; Yamashita et al., 2004; Zdravkovic-Korac et al., 2004; Wu et al., 2012; Rugini et al., 2015). These characteristics have been observed in the regenerated plants of numerous species, such as *R. pseudoacacia* (Han et al., 1993), *A. deliciosa* (Rugini et al., 1991), papaya (Cabrera-Ponce et al., 1996), apple rootstock Jork 9 (Pawlicki-Jullian et al., 2002; Yamashita et al., 2004; Wu et al., 2012), and sweet cherry (Rugini et al., 2015). While the majority of research has concentrated on the initial characteristics of regenerated plants, Mehrotra et al (2013) conducted an investigation into the flowering traits of *R. serpentina*, a shrub characterized by a brief growth cycle (Mehrotra et al., 2013). Their findings revealed that transgenic plants, when compared to nontransformed plants, exhibited normal flowering patterns albeit with a reduced quantity of inflorescences and flowers. Another study conducted in cherries by Rugini et al (2015) tracked *A. rhizogenes*-transformed cherries for 10 years and observed a slight decrease in the number of flowers, while flower morphology, ovule differentiation, and flowering time remained unaltered. The fruit traits of transgenic materials did not change significantly, with the exception of reduced fruit yield due to the decreased number of flowers. The transgenic plants were grafted as rootstocks, reducing the size of the two plants to varying degrees, but fruit quality remained unchanged (Mehrotra et al., 2013). It should be noted that considerable differences in traits exist among the obtained transgenic plants due to strains of *A. rhizogenes* used in transformation and different copy numbers or insertion positions of T-DNA following transformation. Thus, a sufficient number of transgenic plants is required for stable, long-term observation.

The hairy root phenotype of transgenic plants is primarily regulated by *rol* genes. In 1985, White et al. identified and analyzed four *rol* genes, specifically *rolA*, *rolB*, *rolC*, and *rolD*, which play pivotal roles in neoplastic disease induction and hairy root formation (White et al., 1985). Transgenic plants obtained through transformation by *A. rhizogenes* exhibited phenotypes analogous to plants transformed by single or multiple *rol* genes. The *rolB* gene possesses tyrosine phosphatase activity and plays an essential role in root initiation and elongation, contributing significantly to the hairy root phenotype (Filippini et al., 1996). Plants transformed with *rolB* demonstrated typical *A. rhizogenes*-induced phenotypes including increased adventitious roots, loss of apical dominance, and shortened internodes. Examples of such transformed plants include *Pyrus communis*, *Kalanchoe diagremontiana*, and grape rootstocks “Richter 110” (Schmülling et al., 1988; Zhu et al., 2003; Geier et al., 2008). The *rolB* gene specifically upregulates *ARF7* and *ARF19* to promote root initiation in *Nicotiana tabacum* (Bose et al., 2022). The *rolC* is a glucosidase, and its expression correlates with enhanced cytokinin activity in tobacco (Estruch et al., 1991). Plants transformed with *rolC* exhibited dwarfing, dark green leaves, and increased branching, which were associated with reduced gibberellin levels. Furthermore, the introduction of *rolC* into plants such as *P. trifoliata* and *Diospyros kaki* displayed stronger rooting ability (Kaneyoshi and Kobayashi, 1999; Koshita et al., 2002). Similar to *rolB* and *rolC* genes, the *rolA* gene has the ability to induce leaf rooting in *K. diagremontiana* without the need for exogenous hormones (Schmülling et al., 1988). Plants transformed with *rolA* exhibited plant dwarfing and leaf shrinkage, which were associated with decreased gibberellin levels in tobacco (Schmülling et al., 1993). The *rolA* gene encodes a DNA-binding protein akin to the HPV-1 E2 DNA-binding protein (Rigden and Carneiro, 1999). The *rolD* gene promotes plant flowering, increases axillary inflorescence formation and elongation, and facilitates adventitious root formation in both tobacco and *Arabidopsis* (Mauro et al., 1996; Falasca et al., 2010). The *rolD* gene encodes ornithine cyclodeaminase, an enzyme that catalyzes the conversion of ornithine to proline (Trovato et al., 2001).

One notable characteristic of Ri plants is their compact plant structure, distinguished by shortened internodes, reduced plant height, increased branching, and enhanced axillary bud growth. These traits hold significant value in breeding of flowers and fruit trees. The Ri phenotype is heritable, with the inserted T-DNA being transmitted through meiosis (Tepfer, 1984) and inherited in a Mendelian dominant manner (Zhan et al., 1988; Christensen et al., 2019). Ri lines of *K. blossfeldiana*, characterized by increased branches, weakened apical dominance, and shortened internodes following backcross separation, have been successfully applied in commercial plant breeding (Christensen et al., 2019). It is widely recognized that rootstocks profoundly influence scion size. In fruit tree breeding, employing Ri plants as rootstocks is a good way to obtain dwarfed plants without compromising fruit quality (Rugini et al., 2015).

Another notable feature of Ri plants is the alteration of root morphology. Upon infection of host plants with *A. rhizogenes*, T-DNA insertion induces cell dedifferentiation, leading to the formation of hairy roots. Hairy roots exhibit characteristics such as rapid growth, a high degree of branching, and oblique development. Ri plants have demonstrated enhanced rooting ability under both greenhouse and field conditions in several plant species (Lambert and Tepfer, 1992; Pawlicki-Jullian et al., 2002; Casanova et al., 2005). This increased rooting ability offers several advantages. Firstly, it can lead to efficient asexual reproduction and improved adaptation to *in vitro* conditions. Secondly, Ri plants with enhanced root development hold significant promise in sustainable plant agriculture, as this can improve water and nutrient management, which can augment plant drought tolerance (Tepfer, 2017). Additionally, increased root biomass particularly benefits plants such that their roots were used for extracting specific metabolites (Mehrotra et al., 2013).

## 5 Conclusion and future perspectives

Over the past three decades, significant progress has been made in the transformation of woody plants by *A. rhizogenes*. Numerous woody plants have been transformed with *A. rhizogenes*, and regenerated plants can be obtained *in vitro* through hairy roots. The direct use of lignified hairy roots to acquire regenerated plants *ex vitro* offers broad prospects for the application of *A. rhizogenes* in woody plants. In addition, the incorporation of genome sequencing will significantly advance theoretical investigations in the field of woody plants. The utilization of diverse binary vectors with *A. rhizogenes* serves as a genetic tool for conducting *in vivo* investigations on gene functionality in woody plants (Bahramnejad et al., 2019).

The typical hairy root symptoms induced by *A. rhizogenes*, including compact plant types and enhanced rooting ability, have been used in flower breeding and rootstock modifications. Genome sequence analysis has revealed that *A. rhizogenes* undergoes horizontal gene transfer to plants, such as *Nicotiana*, *Linaria*, and *Ipomoea* species (White et al., 1983; Matveeva et al., 2012; Kyndt et al., 2015; Quispe-Huamanquispe et al., 2017). This implies that *A. rhizogenes*, during the process of pathogenesis, introduces multiple plasmid-encoded genes into its host through horizontal gene transfer, which occurs as a natural outcome. Currently, plants transformed with wild-type *Agrobacterium* strains are not classified as transgenic species (Lutken et al., 2012), which is advantageous for the application of Ri plants. The predominant natural *A. rhizogenes* strains used are listed in **Table 2**.

**Table 2:** The representative naturally occurring (wild type) strains of *A. rhizogenes* and their plasmids.

Type of opine	Strain	Plasmid	References
Agropine	A4	pArA4a pArA4b = pRiA4 pArA4c	White and Nester, 1980b; Savka et al., 1990
	ATCC 15834	pAr15834a pAr15834b = pRi15834 pAr15834c	White and Nester, 1980b; Savka et al., 1990
	LBA 9402 (NCPPB 1855)	pRi1855	Savka et al., 1990
Mannopine	LBA 9365 = strain 8196	pAr8196a pAr8196b = pRi8196 pAr8196c	Chilton et al., 1982; Savka et al., 1990
Cucumopine	Strain 2659 = K599	pRi2659	Savka et al., 1990
Mikimopine	NIAES 1724	pRi1724	Akira et al., 1990

However, it is imperative to recognize the limitations associated with these applications. To enhance and exploit natural engineering capabilities, one promising avenue lies in the use of CRISPR-mediated base editing, which serves as a catalyst for “engineering the engineer” (Rodrigues et al., 2021). This approach holds promise for improving *A. rhizogenes*, thereby enabling more effective plant transformation and genome editing. According to the National Center for Biotechnology Information (NCBI) database, 96 strains of *A. rhizogenes* exist (https://www.ncbi.nlm.nih.gov/datasets/genome/?taxon=359) as of the search conducted on 26 September 2023. However, only four strains (LBA9402, A4, K599, and CA75/95) have complete genome sequences. The transformation capabilities of these strains vary, necessitating the sequencing and engineering of strain genomes to improve infectivity.

## Author contributions

Conceptualization, XW and BZ. Data curation, WY and GW. Writing—original draft preparation, WY, GW, XW, and BZ. Writing—review and editing, WX, HL, YH, HY, DY, FC, and JH. Supervision, XW and BZ. All authors contributed to the article and approved the submitted version.
